# A review of the recent advances for the in ovo sexing of chicken embryos using optical sensing techniques

**DOI:** 10.1016/j.psj.2023.102906

**Published:** 2023-06-28

**Authors:** Chuanqi Xie, Wensheng Tang, Ce Yang

**Affiliations:** ⁎ State Key Laboratory for Managing Biotic and Chemical Threats to the Quality and Safety of Agro-Products, The Institute of Animal Husbandry and Veterinary Science, Zhejiang Academy of Agricultural Sciences, Hangzhou 310021, China; † Institute of Animal Husbandry and Veterinary Science, Huangyan Bureau of Agriculture and Rural Affairs, Taizhou 318020, China; ‡ Department of Bioproducts and Biosystems Engineering, University of Minnesota, Saint Paul, MN 55108, USA

**Keywords:** chicken embryos, in ovo sexing, optical sensing, models, identification

## Abstract

The culling of day-old male chicks has caused ethical and economic concerns. Traditional approaches for detecting the in ovo sex of chicken embryos involve opening the eggshell and inner membrane, which are destructive, time-consuming, and inefficient. Therefore, noncontact optical sensing techniques have been examined for the in ovo sexing of chicken embryos. Compared with traditional methods, optical sensing can increase determination throughput and frequency for the rapid sexing of chicken embryos. This paper presented a comprehensive review of the different optical sensing techniques used for the in ovo sexing of chicken embryos, including visible and near-infrared (**Vis-NIR**) spectroscopy, hyperspectral imaging, Raman spectroscopy, fluorescence spectroscopy, and machine vision, discussing their advantages and disadvantages. In addition, the latest research regarding different detection algorithms and models for the in ovo sexing of chicken embryos was summarized. Therefore, this paper provides updated information regarding the optical sensing techniques that can be used in the poultry industry and related research.

## INTRODUCTION

Ethical and economic concerns regarding culling day-old male chicks in the poultry industry have emerged. Male birds are identified via manual sexing following hatching and immediately killed. Day-old male chick culling affects 7 billion birds yearly (Krautwald-Junghanns et al., 2018). In Germany, the culling of day-old male chicks has been banned since 2022, and the culling of chicken embryos should be finished at an earlier stage from 2024, ensuring no pain for the embryos. Therefore, identifying and removing male chicks before hatching is a big step forward for animal welfare. It requires rapid, cost-efficient, highly accurate identification techniques that do not affect hatching rates and chick health. It has been reported that the ability of chicken embryos to experience pain starts to develop on day 7 of the incubation period (Aleksandrowicz and Herr, 2015). Thus, the detection should be carried out before pain perception occurs in chicken embryos.

Several approaches have been explored for identifying chicken sex before hatching. Although some of these proved useful, all displayed limitations, and none provided a protocol for rapid and accurate sexing before hatching. For example, Clinton et al. (2016) employed the PCR-free approach to identify chicken sex using molecular sexing assays. However, this strategy was only designed for laboratory conditions. Some promising solutions, such as hormone measurement, require sampling and processing, and volatile identification involves gas chromatography-mass spectrometry (**GC-MS**) measurement (Weissmann et al., 2013; Xiang et al., 2022). However, these methods are unsuitable for the egg-laying industry since they are time-consuming and high-cost. Contrarily, current progress has shown that optical sensing is more flexible for adoption by the layer industry. In addition, optical sensing is recognized as noninvasive to living organisms and does not require chemical and biological tests.

Previous studies involving fluorescence and Raman spectroscopy have shown that a near-infrared (**NIR**) range of around 910 nm can help distinguish between male and female chicken embryos (Galli et al., 2017, 2018). Thus, the in ovo sexing of chicken embryos can be investigated using optical sensing techniques. The potential impact of optical sensing techniques on chicken embryo sex identification is 3-fold. It can 1) prevent the unethical culling of day-old male chicks, reportedly affecting almost 7 billion birds annually, 2) save egg resources and handling costs, and 3) reduce the labor involved in chicken sexing after hatching.

**Figure 1:**
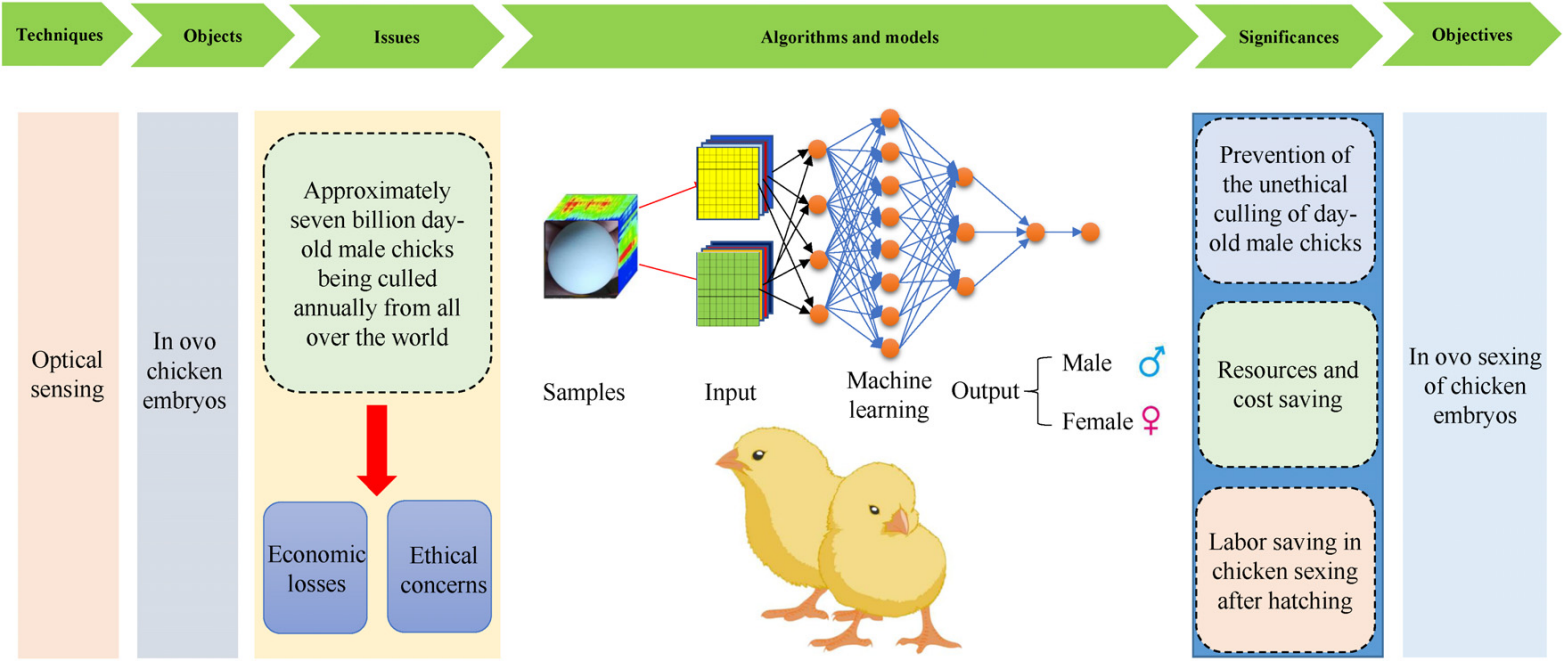
The graphical abstract of this review.

This paper reviews the current situation and development trends of optical sensing techniques combined with machine learning for the in ovo sexing of chicken embryos. The identification approaches are discussed based on techniques such as visible and near-infrared (**Vis-NIR**) spectroscopy, hyperspectral imaging, Raman spectroscopy, fluorescence spectroscopy, and machine vision. The main objectives include 1) discussing recent advances in various remote optical sensing techniques for identifying chicken embryo sex, 2) comparing the advantages and disadvantages of the sensors, and 3) suggesting appropriate detection sensors for specific applications. Figure 1 shows the graphical abstract of this review.

## OPTICAL SENSING TECHNIQUES

Optical sensing techniques such as infrared spectroscopy usually contain sensitive molecular bond vibration information and are suitable for detecting chemical constituents. Thus, the spectral features obtained from optical sensors can be used for identifying chicken embryo sex. The characteristics (e.g., spectral ranges, manufacturers) of the most commonly used optical sensors for the in ovo sexing of chicken embryos are listed in Table 1. These characteristics can be used to determine appropriate sensors for satisfactory identification results. Additionally, the algorithms, models, detection results, features, detection time, advantages, and disadvantages for identifying chicken embryo sex using optical sensors are listed in Table 2, showing the efficacy of optical sensing-based techniques. Furthermore, this paper also discussed the identification accuracy and the effect of the corresponding method on hatching rates. It allows the effective selection of appropriate sensors for research purposes and application in the egg-laying industry.

**Table 1:** The features of the representative sensors used for detecting chicken embryo sex.

Sensors	Schematic diagrams	Spectral ranges	Manufacturers	References
Vis-NIR spectroscopy	/	300–1,145 nm	Zeiss, Oberkochen, Germany	Corion et al. (2022)
Hyperspectral imaging	/	400–1,000 nm	Specim, Oulu, Finland	Göhler et al. (2017)
Raman spectroscopy		150–3,250 cm^−1^	Kaiser Optical Systems Inc., Ann Arbor, MI	Galli et al. (2016)
Fluorescence spectroscopy		795–1,054 nm	Kaiser Optical Systems Inc., Ann Arbor, MI	Galli et al. (2017)
Machine vision	/	/	Basler, Ahrensburg, Germany	Zhu et al. (2021)

**Table 2:** The optical sensing-based techniques for chicken embryo sexing.

Techniques	Algorithms and models	Results	Features	Detection time	Advantages	Disadvantages	References
Vis-NIR spectroscopy	LDA	The DNA content in the male blastoderm cells is about 2% higher than in the female cells	DNA	Before incubation	Accurate and rapid	A hole is opened in the shell, and the blastoderm cells are removed, affecting the hatching rate	Steiner et al. (2011)
	Savitzky-Golay smoothing, derivatives, SNV, mean centering, FiPLS, PLS-DA, etc.	The classification accuracies are 86.49% (day 12), 97.78% (day 13), 99.52% (day 14), and 94.62% (day 18)	Feather color	Day 14	High-throughput, high accuracy, noninvasive, and cost-efficient	The identification time is too late	Corion et al. (2022)
	MSC, CARS, SPA, GA, and ELM	SPA-GA-ELM obtains the best detection accuracy of 87.14%	/	Day 7	Noninvasive	The detection accuracy is low	Zhu et al. (2019)
	Normalization, 2nd der, SNV, MSC, detrend, spectroscopic, PLS-DA, and LDA	The best detection accuracies are 34.37% for PLS-DA and 43.75% for LDA	/	Day 12	Noninvasive	The identification is delayed, and the detection accuracy is too low	Zhang et al. (2022)
Hyperspectral imaging	PCA and LDA	LDA obtains an overall accuracy of nearly 97%	Feather color	Day 14	High accuracy, noninvasive, and the hatchability is not affected	The detection is delayed	Göhler et al. (2017)
	SVM, PLS-DA, and ANN	ANN obtains an accuracy of 82.86%	/	Day 10	Noninvasive	The identification is delayed, and the accuracy is low	Pan et al. (2016)
Raman spectroscopy	LDA	The classification accuracy is 90%	Blood	Day 3.5	Early detection	The eggshell is windowed, and the shell membrane is removed, affecting the hatching rate	Galli et al. (2016)
Fluorescence spectroscopy	SVM and PCA	The best classification accuracy exceeds 90%	Blood	Day 3.5	Early detection	The eggshell is windowed, affecting the hatching rate	Galli et al. (2017)
	Discriminator F_D_	The overall determination accuracy is 96%	Blood	Approximately day 4	Early detection, and the hatching rate is not affected due to the intact inner membrane	The eggshell is opened at the blunt end	Preuße et al. (2023)
Machine vision	GA and BPNN	The average accuracies are 89.74% and 78.58% for double and single hidden layers, respectively	Texture features	Day 4	Early detection and noninvasive	The accuracy is low	Zhu et al. (2021)

## APPLICATIONS

### Vis-NIR Spectroscopy

**Figure 2:**
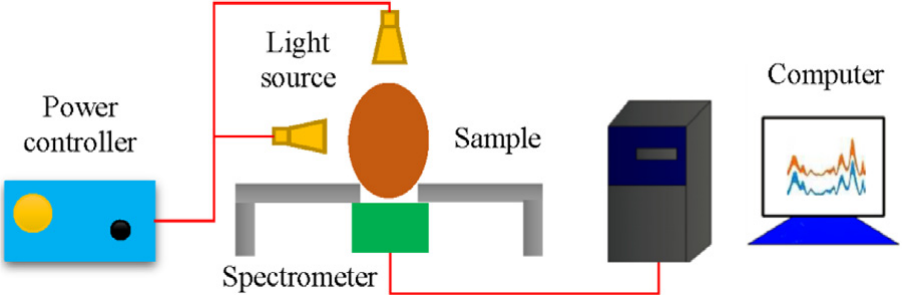
A schematic diagram of spectral reflectance and transmittance information collection.

The schematic diagram of the Vis-NIR spectral reflectance and transmittance information collection is shown in Figure 2, consisting of a spectrometer, a light source, and a powder controller. This system was used to collect spectral information to identify the sex of chicken embryos. Since spectroscopy-based techniques can measure differences in DNA, Fourier transform infrared (**FT-IR**) spectroscopy was used for sex determination by analyzing the spectral features of the germinal disk in freshly laid eggs (Steiner et al., 2011), which consisted of between 40,000 and 60,000 blastoderm cells containing genetic (including gender) information. DNA detection was performed on the unincubated chicken eggs using infrared spectral imaging in a range of 950 to 1,800 cm^−1^. The sex of the chicken eggs was determined based on the differences in the cellular DNA content of the male and female chicks (around 2%). It showed that the blastoderm cells in the males displayed a higher DNA content than the females, which were extracted from the germinal disk. This work demonstrated that FT-IR spectroscopy combined with supervised classification could effectively identify chicken egg sex. However, since this method requires opening the eggshells to allow the optical signal direct access to the embryonic blood vessels, it can result in bacterial contamination or affect embryonic development and hatchability.

In another study, Vis-NIR spectroscopy (300–1,145 nm) was used for the gender identification of chicken eggs (Corion et al., 2022). The spectral signals of the eggs were collected on day 8-14 and day 18 of incubation to investigate the effect of the incubation time on sex identification. Partial least squares-discriminant analysis (**PLS-DA**) models were built for classification, and the variables were selected via forward interval partial least squares (**FiPLS**). The accuracy was 97.78% on day 13, increasing to 99.52% on day 14, while it was 86.49% on day 12. The lower accuracy of 94.62% obtained on day 18 was attributed to the strong light attenuation by the growing chicken embryos. Furthermore, 749 to 861 nm was sufficient for detecting the sex of the chicken eggs at an accuracy of 98.46%. This study provided a new approach for the high-throughput and cost-efficient sex detection of chicken embryos. However, the identification time in this study is too late.

Zhu et al. (2019) used UV-Vis-NIR transmission spectroscopy (360–1,000 nm) to identify chicken embryo sex. The classification accuracies of the extreme learning machine (**ELM**) models were compared regarding different placement methods (vertical and horizontal) and incubation periods. The results showed that vertical placement on day 7 yielded the best identification accuracy. Wavelengths between 360 and 780 nm were used for further analysis since they provided the best classification results (84.29%) among several spectral ranges. Multiplicative scatter correction (**MSC**) was performed to remove noise while competitive adaptive reweighted sampling (**CARS**), the successive projection algorithm (**SPA**), and the genetic algorithm (**GA**) were used to screen the characteristic wavebands. Finally, the best result was obtained via SPA-GA-ELM with a classification accuracy of 87.14% in the prediction set, providing a noninvasive method for identifying chicken embryo sex. However, the classification accuracy was too low for industrial applications. Zhang et al. (2022) identified chicken embryo sex using Vis-NIR spectroscopy (345–1,041 nm), discussing the identification results of different preprocessing methods (e.g., normalize, the second derivative [**2nd der**], standard normal variate [**SNV**], MSC, and detrend) and data collection locations (blunt end, sharp end, and the equator). This study found that preprocessing the eggs on day 12 of incubation yielded good identification results, while combining the blunt end location with the detrend achieved the best classification accuracy (43.75% for linear discriminant analysis (LDA)). Although it provided a new direction for the noninvasive sexing of chicken embryos, the identification was delayed, and the detection accuracy was too low.

In ovo sexing of chicken embryos using spectroscopy techniques can also be seen in the patents. For example, in the patent US20120058052A1, the noninvasive method and apparatus for in ovo sexing of avian species were invented. In this invention, the wavelength of the excitation light and/or the emission light was preferably selected at 700 to 2,000 nm, preferably 700 to 1,500 nm, more preferably 700 to 1,000 nm, or 610 to 630 nm. In another patent (US20130044210A1), a neural network algorithm based on spectroscopy (300–2,500 nm) was used to compare the spectrum of a test egg against a spectral library. This method can detect the gender of the chicken eggs with greater than 75% reliability on day 12 after laying. Furthermore, Terahertz spectroscopy was carried out for the in ovo sexing of chicken embryos. For example, in the patent US20210212296A1, the inventors developed a system for the in ovo sexing of avian embryos, including a sampling apparatus, an electromagnetic radiation transmitter, and a detector. In this patent, the sampling apparatus comprises a vacuum source, a gas collection device, and a membrane. The sampling apparatus directs the gas captured from the egg vicinity toward the membrane. Finally, the membrane is put in the electromagnetic radiation emitted by the transmitter, producing a spectrum for detecting the egg gender.

### Hyperspectral Imaging

**Figure 3:**
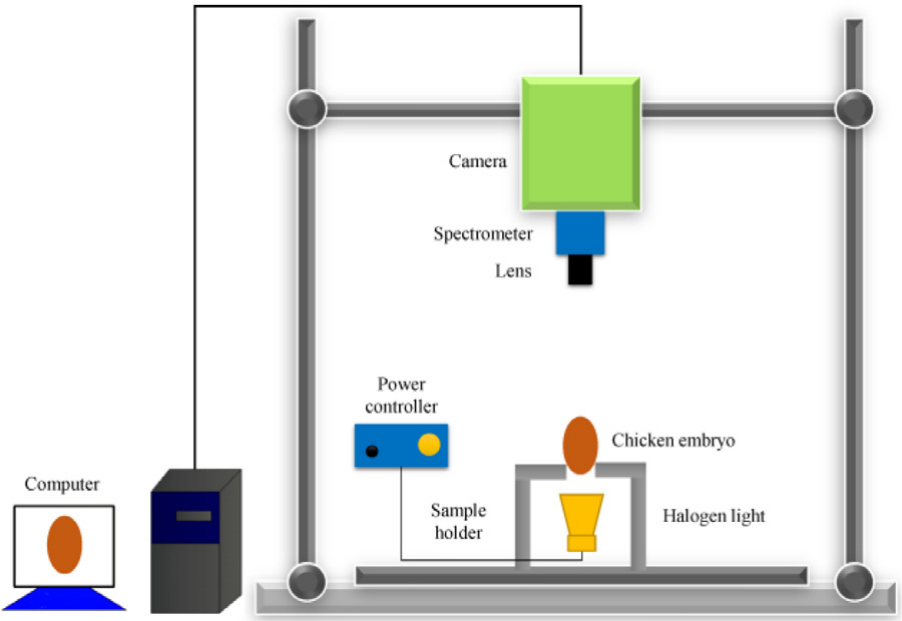
The hyperspectral imaging system for chicken embryo image collection.

The hyperspectral imaging system (Figure 3) simultaneously provides spectral and imaging features. After image collection, the raw hyperspectral image is calibrated according to Eq. (1). Each pixel in the hyperspectral image provides continuous spectral information, thus outperforming Vis-NIR spectroscopy. However, data collection and analysis via hyperspectral imaging are more complicated.

(1) $$I_{calibrated}=\frac{I_{r\text{aw}}-I_{dark}}{I_{white}-I_{dark}}$$

Since hyperspectral imaging provides potentially helpful spectral information and allows pixel-scale image analysis to identify blood spots and vessels in the eggs automatically, it is used for the in ovo sexing of chicken embryos. Göhler et al. (2017) determined chicken embryo sex via the pattern analysis of hyperspectral images. A hyperspectral imaging system at 400 to 1,000 nm was used to collect images of chicken embryos on day 11-14 of incubation. Then, principal component analysis (**PCA**) was performed to obtain independent variables to resolve the high collinearity among the spectral data inconsistencies. LDA was used to classify the male and female chicken embryos. An overall identification accuracy of nearly 97% was obtained for the chicken embryo sex on day 14 of incubation. Moreover, the classification accuracy increased with the incubation time (day 11–14). This study also confirmed that the nondestructive detection via hyperspectral imaging did not affect the hatchability. However, even though the overall accuracy was high, the detection was delayed, causing pain for chicken embryos and economic losses. Another study only achieved 82.86% accuracy in predicting chicken embryo sex on day 10 of incubation using an artificial neural network (**ANN**) and hyperspectral imaging (Pan et al., 2016). The detection accuracy was too low for large-scale industrial applications, and the identification was delayed. Several additional limitations were evident in the experimental setup, such as insufficient backlighting for transmittance imaging and small regions of interest (**ROI**) in the samples for data analysis. Therefore, there is still a potential for accurately predicting chicken sex via hyperspectral imaging by adjusting the light source and utilizing the image of the entire egg as the ROI for data analysis.

### Raman Spectroscopy

Raman spectroscopy can extract information regarding molecular structures and compositions in samples, covering several vital vibrational modes (Lohumi et al., 2017; Joshi et al., 2020). Therefore, this technique was also employed for the sex identification of chicken embryos.

**Figure 4:**
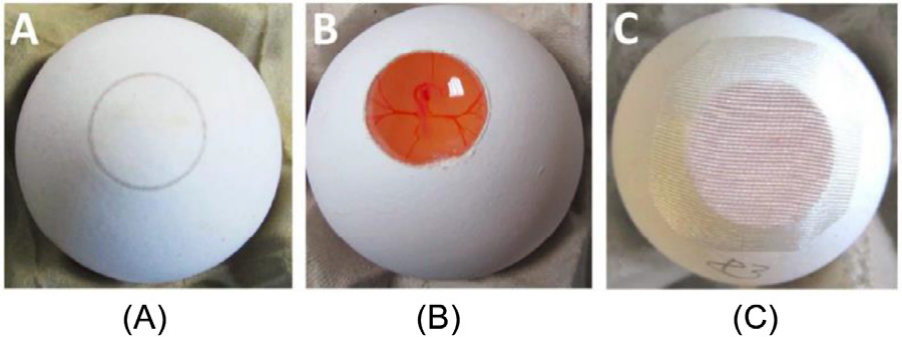
(A) A precut in the shell via laser, (B) a shell window was opened on day 3.5 for Raman spectral collection, and (C) a closed eggshell using biocompatible adhesive tape (Galli et al., 2016).

By opening a 15 mm hole using a CO_2_ laser cutter (Figure 4A) and using a NIR excitation wavelength of 785 nm, Raman spectroscopy allowed the in ovo sexing of chicken embryos according to the spectral features of the germinal or blood cells on day 3.5 of incubation (Galli et al., 2016). The shell windows and membrane were opened on day 3.5 (Figure 4B). After Raman spectral feature collection, the eggshell windows were closed using biocompatible adhesive tape for subsequent hatching (Figure 4C). Sexing identification accuracy of 90% was obtained based on the spectral features of the circulating blood. The results demonstrated that the Raman spectroscopy data of the embryonic blood could be used for the in ovo sexing of chicken embryos on day 3.5 of incubation. As an optical technique, the Raman spectral fingerprint of embryonic blood can be obtained in seconds, providing immediate sex information. However, this method also requires removing the shell window and the membrane, which reduces the hatching rate. Therefore, the hatching process (59 eggs with open shell windows for Raman spectroscopy measurement and 30 control eggs) was investigated experimentally, yielding 81 and 92% hatching rates, respectively.

### Fluorescence Spectroscopy

**Figure 5:**
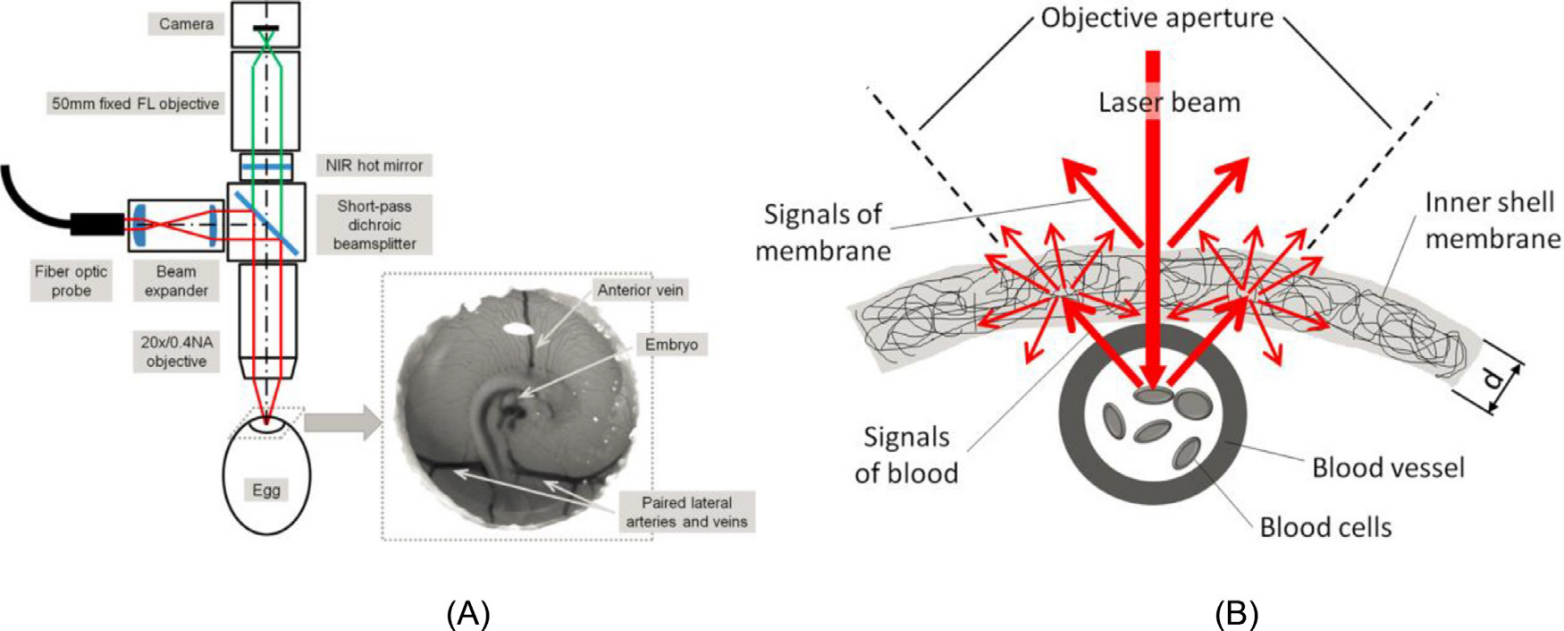
(A) A schematic diagram of the microscopy system for in ovo spectra measurement (Galli et al., 2017), and (B) the scattering effects of the inner shell membrane (Galli et al., 2018).

The schematic diagrams of the microscopy system for the in ovo spectral measurement and the scattering effects of the inner shell membrane are shown in Figure 5A and B. The detection principle of fluorescence spectroscopy involves using a laser to illuminate an object. The fluorescence, which is correlated with the internal organizational structure of the object, will be excited. Therefore, this technique performed well in identifying chicken embryo sex. Galli et al. (2017) identified chicken embryo sex using fluorescence spectroscopy and a NIR laser at 785 nm. The fluorescence spectral differences related to sex were evident on day 3.5. PCA showed there is a specific fluorescence waveband at 910 nm for the blood of male chicken embryos. Support vector machine (SVM) yielded the best accuracy of over 90% based on fluorescence and Raman scattering. Furthermore, Galli et al. (2018) used fluorescence and Raman spectroscopy to identify chicken egg sex via sensing through the shell membrane. This study revealed that the blood of male chicken embryos presented a higher fluorescence intensity than female chicken embryos, and PCA indicated the presence of different fluorescence spectral shapes at around 910 nm. An overall identification accuracy of 93% was obtained via the supervised classification of the spectral features. Since Raman and fluorescence spectroscopy could perfuse the extraembryonic vessels with the inner shell membrane intact, the hatching rate was not affected (96% hatching rate in this study). Therefore, these findings revealed that the negative effect on the hatching rate can be avoided by keeping the inner shell membrane intact.

**Figure 6:**
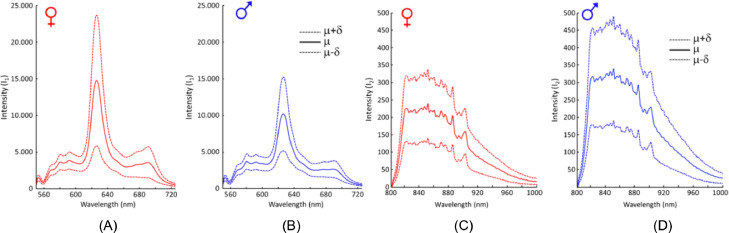
The mean spectra (μ) and standard deviation (δ) of the in ovo fluorescence of the chicken embryos: (A) female, (B) male, (C) female, and (D) male. The excitation wavelengths were 532 nm for (A and B) and 785 nm for (C and D) (Preuße et al., 2023).

Preuße et al. (2023) employed 2-wavelength fluorescence spectroscopy to identify chicken embryo sex. The fluorescence was excited at wavelengths of 532 nm and 785 nm. The mean spectra (**μ**) and standard deviation (**δ**) of the fluorescence at these wavelengths are shown in Figure 6A–D. Female embryos yielded a higher average fluorescence intensity than male embryos at 628 nm, while the opposite was evident at 800 to 1,000 nm. These results indicated that the fluorescence intensity was related to the gender information, while the differences were caused by embryonic hemoglobin synthesis, obtaining an overall classification accuracy of 96%. In this study, the eggshells were opened at the blunt end, leaving the inner shell membrane intact for data collection. Thus, this technique did not affect the hatching rate (in ovo sexing: 96.3% vs. the control: 96.1%).

### Machine Vision

Machine vision images were obtained to detect the gender of chicken eggs (Zhu et al., 2021). Two batches of chicken eggs (186 and 180) were used for image collection on day 3, day 4, day 5, day 6, day 8, and day 10 of incubation. The chicken embryo images collected on day 4 were used as the basis for sex identification. In this study, the image features were extracted based on the gray level co-occurrence matrix (**GLCM**), fractal dimension (**FD**), gray level histogram statistics (**GLHS**), and geometric characteristics (**GC**). Then, 4 GLCM features (energy, contrast, correlation, and entropy), 1 FD feature, 5 GLHS features (mean, standard deviation, smoothness, third-order moment, and consistency), and 1 GC feature (shape index) were obtained to describe the blood vessel characteristics. Finally, the average accuracies in the prediction of genetic algorithm-backpropagation neural network (GA-BPNN) were 89.74% and 78.58%, with double and single hidden layers, respectively. Therefore, optimizing the initial weights and thresholds of the BPNN using GA and adding a hidden layer could improve the detection accuracy. This study confirmed that machine vision was also effective for chicken embryo sex identification during the early stage.

## CONCLUSIONS

Due to the ethical and economic concerns regarding the culling of day-old male chicks, this review explores the feasibility of optical sensing techniques for chicken embryo sexing before hatching. The different optical sensing techniques for the in ovo sexing of chicken embryos are discussed. Vis-NIR spectroscopy, especially at around a wavelength of 910 nm, can classify male and female chicken embryos based on spectral features. The imaging information is also effective for identifying chicken embryo sex based on texture features, such as energy, contrast, correlation, and entropy. Hyperspectral imaging is useful for detecting chicken embryo sex since each pixel of a hyperspectral image spans the entire spectral range. Based on optical sensing techniques, male chicken eggs can be identified immediately. This procedure is usually performed before the pain perception development of the chicken embryo, which is more acceptable than the unethical culling of day-old male chicks, improving animal welfare. The detection time is significant since the identification should be carried out before pain occurs. Thus, Raman spectroscopy, fluorescence spectroscopy, and machine vision show the best performance since they could detect the sex of chicken embryos during the early stage. After chicken embryo sexing, the male eggs can be used in the food industry, reducing economic losses. These identification techniques may be suitable for commercial application in chick hatcheries.

However, the detection accuracy is usually lower than traditional methods. Furthermore, the eggshell presents a challenge during optical sensing analysis since optics access represents the primary approach for sex determination using spectroscopy. The eggshell must be opened to address this problem, usually affecting the hatching rate. Consequently, selecting appropriate optical sensing techniques for identifying chicken embryo gender is critical. Additionally, more useful features (e.g., sensitive wavebands) and advanced algorithms must be developed to improve the detection results. This review may provide references for the large-scale in ovo sexing of chicken embryos in the poultry industry during the early stage.

In future studies, genome editing is also a trend for the in ovo sexing of chicken embryos. The Z chromosome of female chicken can be gene-edited, and only the W chromosome will be transferred to the chicken embryos. Finally, only female chicken embryos (Z and W chromosomes) can be developed successfully.

## Appendix Supplementary materials

Image, application 1
