# Supplementary material for: A review of the recent advances for the in ovo sexing of chicken embryos using optical sensing techniques
Source: Poult Sci. 2023 Jun 28;102(10):102906. doi: 10.1016/j.psj.2023.102906 (PMC10393812; doi:10.1016/j.psj.2023.102906)
Supplement: Supplementary file 1 [file mmc1.docx]

Highlights

- Optical sensing techniques for the in ovo sexing of chicken embryos were reviewed.
- The advantages and disadvantage of the sensors were discussed.
- It provides references for sexing of chicken embryos in the poultry industry.
